# Orthotropic Laminated Open-cell Frameworks Retaining Strong Auxeticity under Large Uniaxial Loading

**DOI:** 10.1038/srep39816

**Published:** 2017-01-04

**Authors:** Hiro Tanaka, Kaito Suga, Naoki Iwata, Yoji Shibutani

**Affiliations:** 1 Department of Mechanical Engineering, Osaka University, 2-1 Yamadaoka, Suita, Osaka 565-0871, Japan

## Abstract

Anisotropic materials form inside living tissue and are widely applied in engineered structures, where sophisticated structural and functional design principles are essential to employing these materials. This paper presents a candidate laminated open-cell framework, which is an anisotropic material that shows remarkable mechanical performance. Using additive manufacturing, artificial frameworks are fabricated by lamination of in-plane orthotropic microstructures made of elbowed beam and column members; this fabricated structure features orthogonal anisotropy in three-dimensional space. Uniaxial loading tests reveal strong auxeticity (high negative Poisson’s ratios) in the out-of-plane direction, which is retained reproducibly up to the nonlinear elastic region, and is equal under tensile and compressive loading. Finite element simulations support the observed auxetic behaviors for a unit cell in the periodic framework, which preserve the theoretical elastic properties of an orthogonal solid. These findings open the possibility of conceptual materials design based on geometry.

A wide variety of cellular and laminated composite solids, which are often observed in the internal microstructures of natural materials, can be characterized according to their spatial frameworks and hierarchical structure. The spatial framework is represented by geometrical divisions formed among different spatial regions and types of material, including soft and hard/brittle materials, at the same scale level^1^,^2^. The hierarchical structure is composed of materials or structures that have different characteristic lengths on multiple scales^3^,^4^,^5^,^6^,^7^. Throughout millions of years of evolution, biological systems under constrained conditions, such as the limitations of the environmental resources and/or their mobility, have developed well-defined microstructures with anisotropic materials. Many of these structures show superior mechanical properties, such as the maximized load capacities of marine organisms^7^,^8^,^9^,^10^,^11^. In contrast, the rapid progress of material processing technology has enabled the design and fabrication of man-made structures, inspired by nature and based on scientific background^12^,^13^,^14^,^15^,^16^. Although may artificial materials have yet to achieve the robustness and multi-functionality of biologically refined materials, artificial systems hold potential for exceptional performances.

Auxetic deformation, with a negative Poisson’s ratio, is an abnormal mechanical characteristic, which is often attributed to microstructures that feature local rotation of some components^17^,^18^. The mechanisms and fabrication of auxetic microstructures has been extensively studied^19^,^20^,^21^,^22^,^23^,^24^,^25^,^26^. Considering the shift in technology towards designing and applying functional materials across a wide range of length-scales, our study focuses on reproducibly achieving practical man-made materials with auxeticity and other unique functionalities.

Poisson’s ratio indicates the ratio of transverse contraction vs. longitudinal stretching. Materials with negative Poisson’s ratios laterally expand when stretching, which has been termed *auxetic behavior* by Evans *et al*.^27^. A variety of auxetic materials, structures, mechanisms and related systems have been reported^17^,^28^,^29^. For example, an array of rotating rigid units can induce auxetic behavior, and this is one of the fundamental auxetic mechanisms according to some background on geometry^30^,^31^,^32^,^33^,^34^,^35^,^36^,^37^. Many auxetic cellular and laminated composite solids can be designed from considerations of geometry and rotation^37^,^38^,^39^,^40^,^41^,^42^,^43^,^44^.

The Poisson’s ratio of three-dimensional isotropic materials has limits of 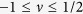 based on the assumptions of classical elasticity theory^45^,^46^. However, an orthotropic material can theoretically exhibit the necessary condition 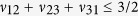 by considering its stability under hydraulic pressure^45^, which means that the lower bound of Poisson’s ratios cannot be expressed as an explicit formula. Indeed, advanced orthotropic microstructures with high negative Poisson’s ratio (below −1) have been created. Some examples include, tethered nodule‒fibril models^47^, fiber network mats^48^,^49^, compliant cellular models^50^, slit perforation models^51^, and double arrowheads^52^. Nevertheless, few real systems that exhibit high negative Poisson’s ratios under both tensile and compressive loads have been reported. It remains unclear whether these orthotropic materials can physically maintain a high negative Poisson’s ratio in a reproducible fashion. To realize the integrity of orthotropic materials with strong auxeticity, here we design a novel laminated open-cell framework with orthogonal anisotropy. We also discuss the auxetic behavior of the fabricated structural units by uniaxial loading tests and numerical simulations.

## Results

**Figure 1:**
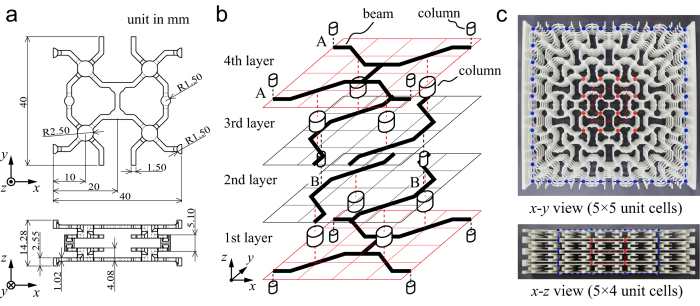
Overview of the proposed cellular frameworks; (**a**) top and side views of a unit cell, (**b**) schematic of the multilayered construction, (**c**) photograph of structure fabricated by additive manufacturing, comprising 5 × 5 × 4 unit cells in *x, y, z* coordinates with components made of either nylon or ABS resins. In the *x*-*y* view, the red and blue markers correspond to a unit cell and 4.5 × 4.5 unit cells, respectively. In the *x*-*z* view, the red and blue markers correspond to 1 × 2 unit cells and 3 × 4 unit cells, respectively.

The structure presented here is an open-cell framework and consists only of beam-like components made of the same material. Figure 1 shows an overview of the proposed structure, which was fabricated by an additive manufacturing technique (3D printing). The in-plane geometry of the cellular structure was composed of four-coordinate circular nodes and 45°-double-elbowed beams with a rectangular cross-section. The beams were repetitively connected to each other. In the column normal to this plane, adjacent in-plane structures were connected in a laminate fashion. Top and side views of a unit cell of the structure are shown in Fig. 1(a). A Cartesian coordinate system was used for the in-plane *x*-*y* and out-of-plane *x*-*z* coordinates. In the center of the top view of the unit cell a single straight beam can be seen (aligned in the *y*-axial direction), which results in the in-plane orthotropic shape. These reinforced beam members play a major role in the out-of-plane auxetic deformation as discussed later. Figure 1(b) illustrates the multilayered structure in a unit cell. For laminate processing in the vertical direction (+*z*-direction), the first-layer, indicated by the red grid, is connected to a pair of beam members with the rack-shaped pattern parallel to the *x*-axis. The second-layer, indicated by the black grids, includes the pair of beam members, both with their rack-shaped pattern parallel to the *y*-axis. In the first layer only, the two beam members are bridged by a single straight beam along the *y*-axis. The first- and second-layers are connected by four columns located at the center of each quadrant of the unit cell. The upper and lower parts of the columns act as a four-coordinate node with a circular cross-section, with the four beam members rigidly connected. The third- and fourth-layers form a mirror reflection of the structure in the *z*-axial direction and are thus replicas of the second- and first-layers, respectively. The first- and fourth-layers, and the second- and third-layers are connected by two columns per unit cell in a periodic fashion, located at points A and B, respectively, as shown in Fig. 1(b).

The test specimens for the cellular structure were manufactured with two types of 3D-printing machines: one was fabricated by laser sintering modeling with nylon resin; the second was fabricated by fused deposition modeling with acrylonitrile butadiene styrene (ABS) resin. Full details of the equipment and conditions can be found in the Methods and Supplementary Information. The material properties of nylon and ABS resin are summarized in Table 1. While nylon resin is a highly ductile material, ABS resin has a brittle nature. Hence, after forming the ABS structures, we immersed them twice in commercial silicone oil to enhance their fracture resistance. The toughness of the treated ABS material more than doubled in the tensile experiments, as determined from comparison of pipe specimens (see Supplementary Fig. S1). Figure 1(c) shows a photograph of a real test specimen with 5 × 5 × 4 unit cells made of nylon resin. The dimensions of the structural units without side bars were 200 × 200 × 57.12 mm^3^. Both ends of the specimens, in the *x*-axial direction, were modified for attachment to the jig of the tensile loading equipment. For compression, the platen was used to press both sides of the structure directly and without any special attachment (see Supplementary Fig. S2). In single *x-y* or *x-z* planes, some nodes were colored as characteristic points to be traced by image processing software to assess the cell deformation during loading tests. Groups of red- or blue-colored nodes correspond to in- and out-of-plane deformations of the inner or outer cells in both surfaces, respectively.

Using the test specimens described above, we examined the quasi-static tensile and compressive properties of the proposed cellular structures. In all the experiments, the displacement speeds were 1 mm/min (5.0 × 10^−3^ strain/min). Full details of the universal testing machine and test conditions are described in the Methods.

**Figure 2:**
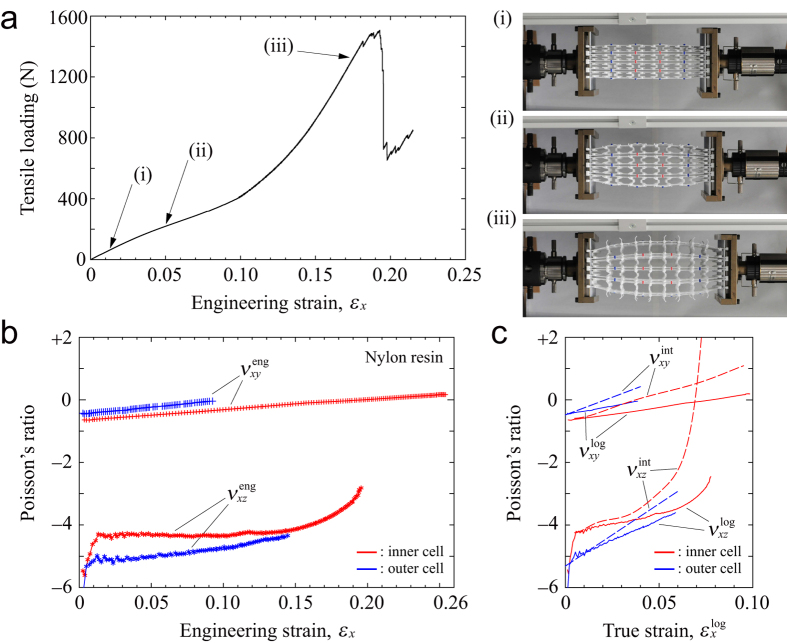
Tensile test for the nylon resin cellular framework: (**a**) load vs. strain curve, where the points (i)–(iii) corresponds to the inset photographs of auxetic deformation; (**b**) measured in- and out-of-plane Poisson’s ratio curves as a function of engineering strain for the inner and outer cells; (**c**) two types of Poisson’s ratios calculated by true strain or instantaneous true strain.

The uniaxial testing results for nylon resin are summarized in Fig. 2. Figure 2(a) shows the load–strain curve under tensile deformation up to the fracture point. The nylon resin gives the structure superior ductile behavior; the fracture onset was at a strain of 19.18% and the maximum tensile load was 1506.7 N. The tensile curve displays the typical mechanical properties of an elastomeric honeycomb or foam^1^. Initially, the specimens showed a linear elastic relationship, where the modulus was determined by bending of the beam members. At a strain greater than 10% the tangent stiffness increased monotonically because stretching dominated the deformation rather than bending by alignment of beam and column components in the tensile direction. The inset images of deformation snapshots, corresponding to points (i)‒(iii), indicated in the tensile curve. These images show that the structure became barreled and increased in thickness at higher tensile loads. After exceeding the maximum tensile load, cleavage cracks appeared with microscopic rapture events in the beam members.

The tensile test gives the measured Poisson’s ratios, 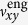 and 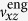 as a function of engineering strain *ε*_*x*_ for the in- and out-of-plane deformations, which were calculated by analyzing the image sequence acquired from the top and side views during deformation (see the Supplementary Information). As shown in Fig. 2(b), the values of 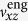 for the inner cell, indicated by red, held at around −4 even at large deformations. Conversely, the values of 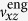 for the outer cell (blue curve) were initially below −4 because the free surfaces at the upper and lower sides in the out-of-plane direction allowed the outer cells to be deflected more largely, but gradually increased with increasing *ε*_*x*_. This elastic behavior with high negative Poisson’s ratios is attributed to three-dimensional rigid rotation of the four-coordinate circular nodes as follows: each column node is located at the center of a unit cell quadrant and under an applied load the cell expands in the plane owing to contra rotations of adjacent nodes involved with bending of the connected beam members^39^. An analogous mechanism has been reported for rotation of rigid square components^30^. However, here the vertically reinforced beam prevented the cells from expanding freely, which resulted in compression of pairs of the elbowed beams parallel to the vertically reinforced beam. As a result, out-of-plane rotations of the four-coordinate circular nodes were triggered.

As described above, the nodal rotations also caused in-plane auxetic behavior. Indeed, the 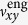-curves in Fig. 2(b) showed a negative Poisson’s ratio at the initial phase although the values of 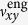 gradually increased for both the inner and outer cells, eventually becoming positive. Note that there was a slight difference in 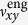 between the inner and outer cells because the outer cells consisted of imperfect 5 × 5 unit cells, such that the Poisson’s ratios were underestimated, as shown in Fig. 1c (*x-y* view). At a high strain of around 10% the large deflection of the beam members over the free surfaces prevented our image processing from tracing the characteristic points of the outer cells (see Supplementary Movie S1 and S2). The 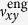
- and 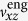-curves for the outer cells (blue curves) were truncated for comparison with those of the inner cells (red curves). Note that the measured engineering strain of the in-plane Poisson’s ratio for the inner cell achieved around 25% because of the large surface extension.

The conventional formulation of the Poisson’s ratio, defined by engineering strain, is misleading for highly nonlinear elastic materials^53^. Figure 2(c) shows the two types of Poisson’s ratio, 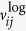 and 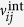, which are defined by true strain and instantaneous true strain, respectively. The measured instantaneous true strain is sensitive to experimental noise which depends on the form of the dimension data^53^. Hence in our experiments, the 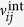-curves (the dashed curves) were obtained by fitting the averaged data (see Fig. S5). The calculated 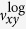
- and 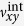-curves for the inner cell increased linearly with increasing true strain whereas the 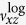
- and 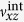-curves for the inner cell increased exponentially in the final phase. In particular, the 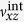-curve (the red dashed curve) goes far beyond zero, which means that the structure no longer exhibited auxetic behavior with respect to its current configuration. Thus, these results illustrate how stretching-dominated deformation relates to component orientations under large deformations.

**Figure 3:**
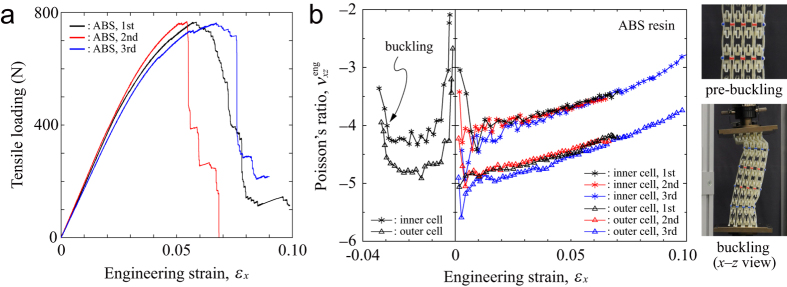
Tensile and compressive tests for the cellular frameworks made of ABS resin: (**a**) load vs. strain curves for the three different structures; (**b**) measured in- and out-of-plane Poisson’s ratio curves as a function of engineering strain on the three tensile tests and one compression test. Two inset photographs show the profiles of pre-buckling and buckling in the *x*-*z* views. Note that the adjacent cells are in contact with each other just prior to buckling.

The results of tensile and compressive tests for structures made of ABS resin are shown in Fig. 3 and Supplementary Movies S3 and S4. We conducted each tensile test three times and Fig. 3(a) shows the three curves of the relationship between load and engineering strain. The nonlinear elastoplastic behavior (i.e., the strain at the maximum load or the breaking elongation) were largely different in the respective curves. These differences might have resulted from specific features of the resin failure probabilities; however, the levels of the maximum loading were consistent with each other. Furthermore, the three curves of Poisson’s ratio with respect to engineering strain showed very similar behavior (see Fig. 3(b)). The Poisson’s ratios started from around −4 and −5 for the inner and outer cells, respectively, and gradually increased to their fracture points. We also conducted the compressive test once and two curves showing the Poisson’s ratios for the inner and outer cells are shown in Fig. 3(b). Initially the Poisson’s ratio exhibited a constant value of around −4 or −5, which ensured that the Poisson’s ratios were equal under either tensile or compression deformation close to the initially balanced state without loading. Conversely, for the case of high compression the structure buckled at a strain of −0.03 and auxetic behavior disappeared. This unstable mechanism was classified as standard buckling from axial compression to lateral bending. Thus, deflection of the overall structure occurred in the out-of-plane direction against the uniaxial compression. The structure with strong auxeticity promoted buckling owing to shrinking of its cross-section. Pre-buckling deformation proceeded until adjacent cells were in contact with each other (see the insets of Fig. 3(b)).

The finite element model can show the pure periodic behavior of the proposed structural unit, unlike measurements of real structures with finite cell units that include free surfaces and boundary constraints. Full details of the finite element modeling are described in the Supplementary Information. The uniaxial deformation analyses give the elastic properties at a strain of around 0.1%, as listed in Table 2. Here, we define the dimensionless Young’s modulus as *E*_*i*_ = *E*_i_^*^/*E*_s_ (*i* = 1, 2, 3), where *E*_i_^*^ is the structural Young’s modulus subjected to *X*_*i*_-axial force and *E*_s_ is the Young’s modulus of the component solid; the Cartesian coordinates *X*_1_, *X*_2_, *X*_3_ are used in the analysis, which correspond to *x, y, z* in the experiments, respectively. Note that the precise elastic properties are only available when performing the convergent calculations of the finite element model implemented with geometric nonlinearity because the linear analysis cannot clearly reflect out-of-plane deformation when an *X*_2_-axial force is applied to the structure. The calculated Poisson’s ratios, *ν*_12_ and *ν*_13_ agreed well with the experimental results for the nylon resin, as shown in Fig. 2. In addition, *ν*_23_ exhibited a high negative value (−3.955), which was only slightly smaller than that of *ν*_13_. Thus, the structure subjected to the *X*_2_-axial load deformed in a similar out-of-plane manner, with respect to the auxetic behavior under *X*_1_-axial loading. Table 2 also indicates that the Young’s modulus *E*_3_ was very low compared with the other Young’s moduli (*E*_1_ or *E*_2_) according to the theoretical relationship between the elastic moduli and the Poisson’s ratios for an orthotropic solid:


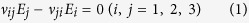


Equation (1) is derived from the symmetry of the compliance tensor of an orthotropic solid^45^,^54^. The numerical elastic properties obtained in Table 2 satisfied the following relationships:


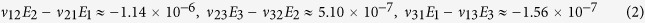


Therefore, the proposed structure sacrifices out-of-plane stiffness to acquire highly negative Poisson’s ratios.

**Figure 4:**
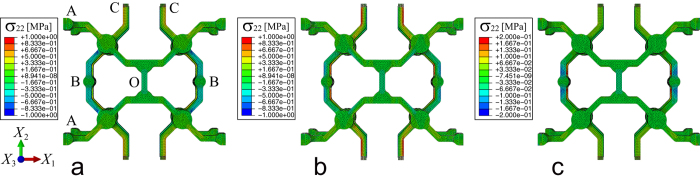
Distribution of *σ*_22_ for a periodic unit cell subjected to a uniaxial tensile load, as calculated by finite element simulations; (**a**) (*F*_1_, *ε*_1_) = (0.696, 0.100) under *X*_1_-axial loading at the side surfaces A, (**b**) (*F*_2_, *ε*_2_) = (0.608, 0.100) under *X*_2_-axial loading at the side surfaces C, and (**c**) (*F*_3_, *ε*_3_) = (0.026, 0.100) under *X*_3_-axial loading at the in-plane surfaces A, where the units are [N] and [%], respectively.

Figure 4 shows the distribution state of *σ*_22_ in the *x*-*y* view of a unit cell under an applied uniaxial tensile force (*F*_1_, *F*_2_ or *F*_3_). The structural deformations when subjected to the three different forces are interpreted as follows. When the structure extends in the *X*_1_-axial direction, i.e., for a tensile force *F*_1_ acting at both edge surfaces A, the out-of-plane deformation occurs mainly by *X*_3_-axial deflection of pairs of the 45°-double-elbowed beams parallel to the *X*_2_-axis (see Fig. 4(a)). This mechanism is triggered by out-of-plane rotations of the four-coordinate nodes, relaxing the compression stress state of the vertically connected beams, as mentioned previously. However, the manner of out-of-plane deformation under *X*_2_-axial tension is slightly different from that of pure *X*_3_-axial deflection of the beams. Figure 4(b) shows that the upper and lower pairs of the vertically connected beams were mostly deflected in the *X*_1_-*X*_2_ plane. Hence, the auxetic deformation in the *X*_3_-axial direction was caused by screw motions of the four-coordinate nodes, each of which was supported at the points B and C via the connecting beams. The screw motion partially includes the in-plane rotation, which explains why *ν*_23_ never achieves the magnitude of *ν*_13_. In contrast to the out-of-plane auxetic behavior, the structural deformation under *X*_3_-axial tension involves *X*_3_-axial local deflections of beam members around the points B, where the nodal rotations are hardly generated (see Fig. 4(c)).

## Discussion and Conclusions

In summary, we designed and built cellular laminated frameworks having orthogonal anisotropy by additive manufacturing. Uniaxial tensile test of a specimen constructed from a ductile material (nylon resin) revealed lateral and perpendicular expansion owing to three-dimensional rotation of component members. The structure maintained a high out-of-plane negative Poisson’s ratio despite large deformations. Furthermore, we performed tensile and compressive tests for a brittle material (ABS resin), which resulted in reproducible auxetic behavior and also displayed equivalent negative Poisson’s ratios against both of the tensile and compressive loads until buckling emerged. After numerical validation of the strong auxeticity with a finite element method we discussed the orthotropic nature of the structure.

The Poisson’s ratio of the proposed structure can be adjusted with a change in the interlayer distance among the four layers of the unit cell. Increasing the height of the column members will reduce the degree of the negative Poisson’s ratio. The addition and removal of the reinforced beam and column members also will cause the Poisson’s ratio of the unit cell to change. By taking advantage of the widely tunable Poisson’s ratios of this structure, we could optimize the hierarchical aggregation of the structural units to control the deformation morphology by small tensile manipulations, while preserving the basic skeleton of the orthotropic structure. For example, some structural units can be transformed into structures having surface asperities and asymmetric structures such as airfoil-like geometries, including the structural union of these geometries of different sizes. Such deformability gives the structural materials unique functionality across multiple length-scales, such as enhanced frictional resistance due to an increase in real contact area^55^,^56^ and improved lifting performance with reduced drag effects^57^,^58^,^59^.

## Methods

### Additive Manufacturing and Post-processing

We designed the proposed framework using 3D-CAD design software (SolidWorks, Dassault Systèms SolidWorks Corp.). Two types of 3D-printing machines, which read CAD-data, were used to fabricate the repetitive cellular structure based on either nylon or ABS resin. The first machine (Formiga P110, EOS GmbH) used a selective laser sintering method with a stacking pitch of 0.10 mm and produced nylon structures with the dimensions 206.0 × 200.0 × 54.5 mm^3^ and a weight of 151.1 g, including jig attachments at both the *x*-axial sides. The second machine (uPrint SE Plus, Stratasys Ltd.) used a fused deposition modeling method with a stacking pitch of 0.254 mm and produced ABS structures with average dimensions of 203.0 × 200.0 × 54.5 mm^3^ and an average weight of 145.9 g including jig attachments at both the *x*-axial sides. To enhance the ductility of the ABS structures, we immersed each specimen twice in commercial silicone oil (Shin-Etsu Silicone Type: KR-251, Shin-Etsu Chemical Co., Ltd.), the solvent of which was composed of 100% toluene. The weight of these structures increased 13–37%, depending on the immersion time and the solvent-to-silicone ratio, as determined by vaporization of toluene.

### Loading Tests and Measurements

We performed tensile or compressive loading tests using a universal testing machine (AG-50KNX, Shimadzu Corp.) equipped with a load cell of 10 kN. Throughout the experiments, the displacement speed of the crosshead was 1 mm/min and the ambient temperature was about 24 °C. The jig attachment was specially designed and processed by Morimitsu Design Co., Ltd. The upper jig attachment connected with the load cell via a universal joint while the lower was fixed to the ground table. The platen was used for pressing without any attachment as for compressive testing (see Supplementary Figure S2). We used two types of common cameras for sequential imaging of the inner and outer cell deformations in parallel with measurements of the load‒displacement curves. A D300 digital camera (Nikon Corp.) with a pixel size of 2848 × 4288 was used to image the *x*-*z* surface side. A NEX-7 (Sony Corp.) with a pixel size of 4000 × 6000 was used to image the *x*-*y* surface side. The distances from the first and second cameras to the object were 2.0 m and 0.5 m, respectively. The images were acquired by both cameras at 15 s intervals, corresponding to a stroke displacement of 0.25 mm per image. We read pixel information of characteristic points from the digital images using the Image Processing Toolbox of Matlab (ver. R2015b, The MathWorks, Inc.), the details of which are summarized in the Supplementary Information.

### Numerical Simulations

Full details of the finite element analyses are described in the Supplementary Information.

## Additional Information

**How to cite this article**: Tanaka, H. *et al*. Orthotropic Laminated Open-cell Frameworks Retaining Strong Auxeticity under Large Uniaxial Loading. *Sci. Rep.* **7**, 39816; doi: 10.1038/srep39816 (2017).

**Publisher's note:** Springer Nature remains neutral with regard to jurisdictional claims in published maps and institutional affiliations.

## Supplementary Material

Supplementary Information

Supplementary Movie S1

Supplementary Movie S2

Supplementary Movie S3

Supplementary Movie S4

## Figures and Tables

**Table 1:** Mechanical properties of the nylon and ABS resins.

	Young’s modulus (MPa)	Tensile strength (MPa)	Fracture strain (%)
nylon^60^	1700	50	20
ABS^61^	2200	31	6

**Table 2:** Calculated elastic properties of the structure under a small tensile strain of around 0.1% according to the finite element simulations with geometric nonlinearity.

*E*_1_^*^/*E*_s_	*E*_2_^*^/*E*_s_	*E*_3_^*^/*E*_s_	*ν*_12_	*ν*_21_	*ν*_23_	*ν*_32_	*ν*_31_	*ν*_13_
7.18 × 10^−4^	6.28 × 10^−4^	9.75 × 10^−6^	−0.604	−0.527	−3.955	−0.062	−0.063	−4.638
