# Supplementary material for: Orthotropic Laminated Open-cell Frameworks Retaining Strong Auxeticity under Large Uniaxial Loading
Source: Sci Rep. 2017 Jan 4;7:39816. doi: 10.1038/srep39816 (PMC5209672; doi:10.1038/srep39816)
Supplement: Supplementary Information [file srep39816-s1.pdf]

Supplementary Information

Orthotropic Laminated Open-cell Frameworks Retaining Strong Auxeticity under Large Uniaxial Loading

Hiro Tanaka[1*], Kaito Suga[1], Naoki Iwata[1], Yoji Shibutani[1]

[1]Department of Mechanical Engineering, Osaka University
2-1 Yamadaoka, Suita, Osaka 565-0871, JAPAN

*Corresponding author, e-mail: htanaka@mech.eng.osaka-u.ac.jp

Supplementary Information:

Supplementary Discussion 1–4, including Supplementary Figures and Tables.

- 1: Fabrication of ABS Specimens Immersed in Silicone Oil

- 2: Design of Jig for Tensile and Compressive Tests

- 3: Image Processing and Definition of Poisson's Ratios

- 4: Finite Element Modeling of a Periodic Unit Cell

References for Supplementary Information

Captions for Supplementary Figure S1–S6

Captions for Supplementary Table S1 and S2

Captions for Supplementary Movie S1–S4

Supplementary Discussion 1: Fabrication of ABS Specimens Immersed in Silicone Oil

We assessed improvements in the brittle ABS specimens after immersion in silicone oil in a prior loading test. Figure S1 summarizes our findings. We fabricated a hollow cylinder (pipe) using a 3D-printing machine (uPrint SE Plus, Stratasys Ltd.), having a cross-section with an outer-diameter of 45 mm and thickness of 1 mm. The pipes had a micro-patterned structure with a pair of rectangular pores alternately arranged in the transverse and vertical directions (see Fig. S1(a)). As shown in Fig. S1(b), we developed a loading test device for the cellular pipe using specially processed jigs, and examined the uniaxial tensile test at a displacement speed of 1 mm/min. Figure S1(c) shows the measured load–stain curves and Table S1 shows the experimental conditions and results. The ductility of structures immersed in silicone oil was enhanced compared with that of untreated structures. The maximum loads and fracture strains of the silicone-oil-treated structures were roughly twice as high as those of the untreated structures.

Thermoplastic polymer materials are in general soluble in various organic solvents; in this case, toluene containing silicone promoted the erosion of the ABS resin components. Such erosion often induces chemical stress cracks inside the material, which result in reduced ductility. However, the fine structures fabricated by 3D-printing machines tend to be composed of low density solids, with properties determined by the in-plane resolution, stacking pitch, and path planning algorithm of the machine. In our experiments, the silicone elastomers might penetrate microscopic pores in the ABS resin specimens at the same time as toluene dissolves into the material. In this way the silicone oil reinforced the structures.

In the main experiments, we also prepared cellular structure specimens made of ABS resin, which were immersed in silicone oil. We have included information on the prepared cellular structures and their loading test results along with those of the structure made of nylon resin in Table S2.

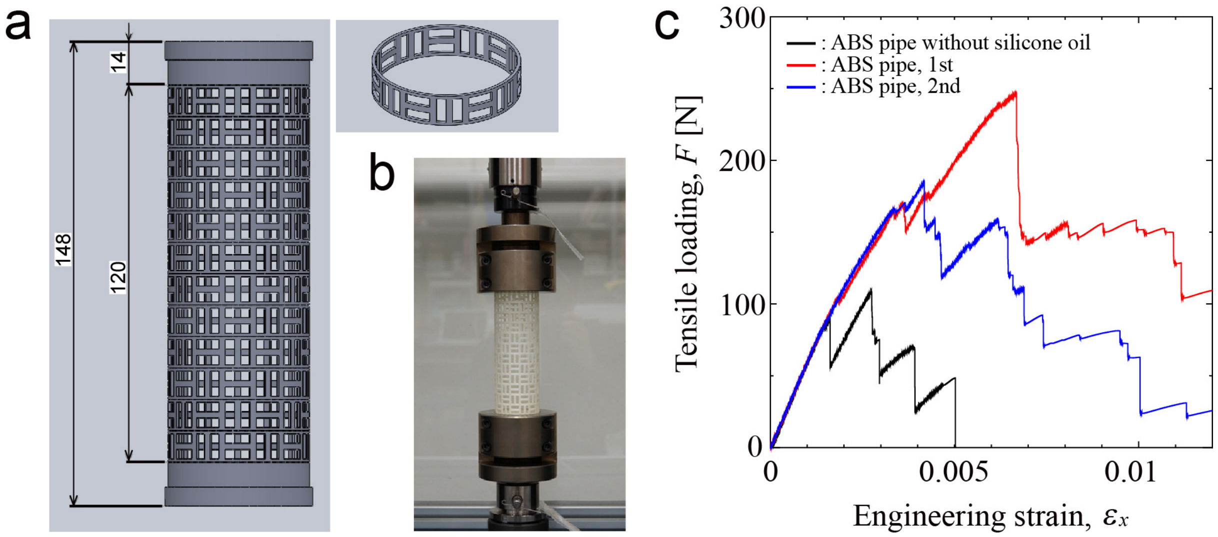

Fig. S1 Uniaxial tensile tests for the ABS microstructure pipes with or without silicone oil; (a) the CAD-data, (b) the uniaxial tensile testing, and (c) load–stain curves under uniaxial extension.

Table S1 Information on as-prepared pipe specimens and test results.

	material	silicone oil	dry weight [g]	final weight [g]	F_{max} [N]	ε_f [%]
pipe #1	ABS	no	57.0	–	108.7	0.28
pipe #2	ABS	yes	57.6	58.6	246.7	0.67
pipe #3	ABS	yes	57.6	59.5	185.0	0.42

Table S2 Information on as-prepared cellular structure specimens and test results.

	material	silicone oil	dry weight [g]	final weight [g]	F_{max} [N]	ε_f [%]
cell #1	nylon	no	151.1	–	1506.7	19.30
cell #2	ABS	yes	146.0	165.7	765.8	5.85
cell #3	ABS	yes	146.0	175.8	767.4	5.44
cell #4 (for compression)	ABS	yes	145.7	185.6	–	–
cell #5	ABS	yes	146.0	203.9	761.4	6.71

Supplementary Discussion 2: Design of Jig for Tensile and Compressive Tests

For precise load testing of the proposed cellular structure, a pair of the specialized steel jigs was manufactured. The upper jig, illustrated in Fig. S2, consisted of one main platen and a pair of jig caps. In the case of tensile loading, the platen and two caps were joined with six bolts, between which ten steel pins were inserted. Each of the pins passed through the side of the specimen so that the tensile forces were transferred to the structure via the pins. Under x-axial uniform tensile displacement the structure was fixed in the z-direction at both the sides because frictional forces acted on the interfaces between the pins and specimen. However, when the perpendicular force driven by the out-of-plane deformation overcame the maximum static frictional force, the side bars were prone to slip in the z-direction (see Movie S1). Specially-processed side bars were welded in-plane with the inside cells, which were fixed in the y-direction at the ends. In the case of compressive loading, a pair of the platens in direct contact with the specimen was used for pressing the ends of the cellular structure. The structure remained fixed to the surface of the two platens until the onset of buckling.

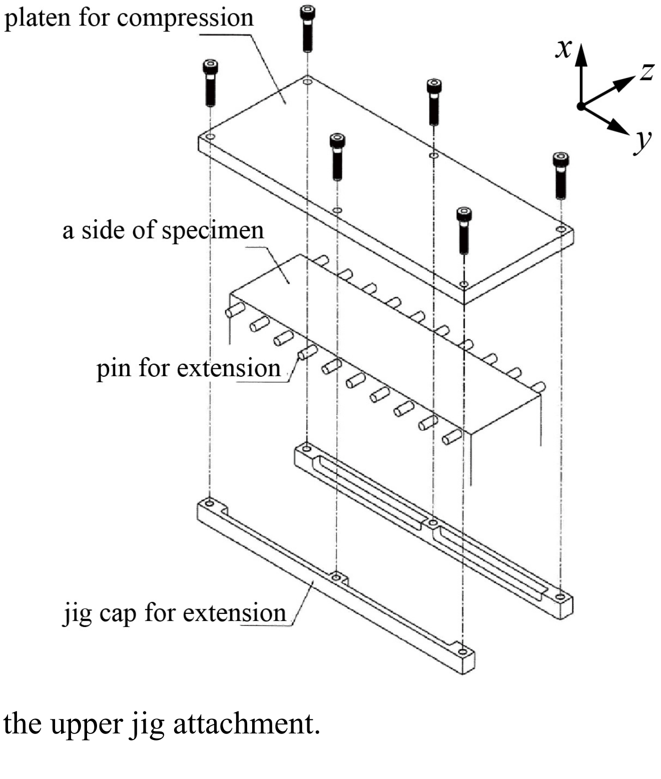

Fig. S2 Schematic of the upper jig attachment.

Supplementary Discussion 3: Image Processing and Definition of Poisson's Ratios

To measure the cell deformation from the photograph sequence during the loading tests, we used conventional image processing, as follows: in the first step, the RGB values of the pixel data in the original image were converted to YCbCr color space. The third and second data groups of the color space, which correspond to red- and blue-difference chroma components, respectively, were selectively extracted (see Fig. S2(i) and (i)'). In the second step, for each of the data sets, a binary image was generated by threshold adjustment after reducing the image noise with Gaussian filtering (see Fig. S2(ii) and (ii)'). In the final step, the centroids of the characteristic points were calculated. We estimated the height and width of the inner and outer cells from the average positions of all the centroids on each cell edge. Note that, for the inner cells of the *x*-*y* surface, the cell lengths were calculated using the center unit cell length that we captured. The four nodal positions were connected with straight lines, each of which was the centroid of the four-point-groups that the sixteen red makers were equally divided into (see Fig. 1(c)).

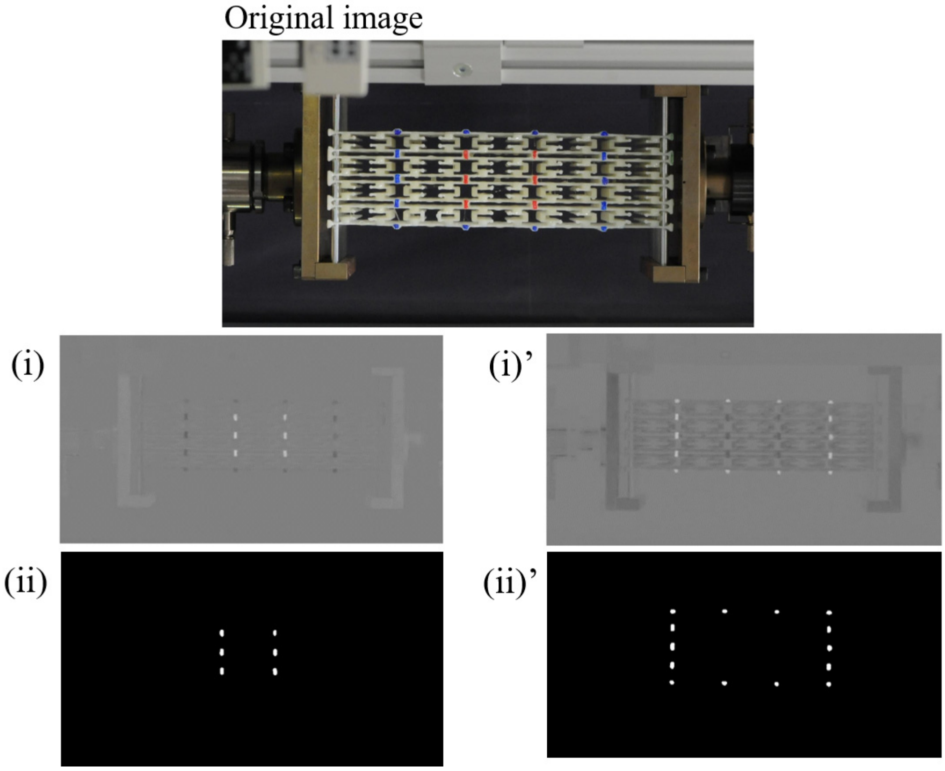

Fig. S3 Example showing the image processing steps for *x*-*z* surface image of ABS structure.

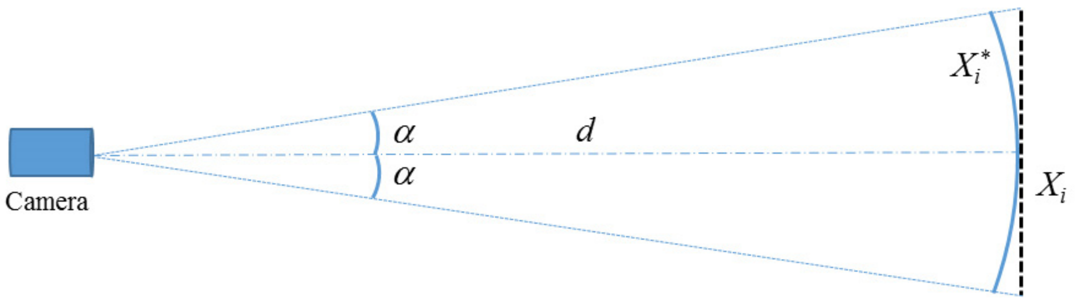

Fig. S4 Schematic of the camera parameters used to correct the dimensions of the specimen.

Assuming a point light source, the measured dimensions obtained from the image data were corrected to the real dimensions of the deformed cells with high accuracy. We ignored errors with respect to lens aberration. Let α and d be the angle and distance from the object, respectively (see Fig. S4). The dimension X_i^* on a camera image can be described as:

$$X_i^* = 2d\alpha\,, \tag{S1}$$

where the subscripts i = 1, 2 and 3 correspond to the coordinates x, y, z in the real space, respectively. From Eq. (S1), the real dimension X_i can be expressed as,

$$X_i = 2d\tan\alpha = 2d\tan\left(\frac{X_i^*}{2d}\right). \tag{S2}$$

Using Eq. (S2), we converted all the cell dimensions from the images during deformation to their real dimensions.

Poisson's ratio is commonly calculated by designating engineering (nominal) strain as ν_{ij}, where the first index refers to axial strain and the second to transverse strain. However, the Poisson's ratio defined by engineering strain is sometime misleading when the targeted structure deforms greatly. Thus, we considered other types of Poisson's ratio [S1].

We defined the specimen dimensions of the n-th image as $X_i^{(n)}$, where $X_i^{(0)}$ indicates the initial configuration. The engineering strain was calculated as follows:

$$\varepsilon_i = \frac{X_i^{(n)} - X_i^{(0)}}{X_i^{(0)}}. \tag{S3}$$

The true strain is then obtained by,

$$\varepsilon_i^{\log} = \int_{X_i^{(0)}}^{X_i^{(n)}} \frac{\delta X_i^{(n)}}{X_i^{(n)}} = \ln\left(1+\varepsilon_i\right) \quad . \tag{S4}$$

Accounting for the current deformation state, the ratio of the extension to the instantaneous length could then be approximated as

$$\varepsilon_i^{\text{int}} = \frac{\delta X_i}{X_i^{(n)}} \approx \frac{X_i^{(n)} - X_i^{(n-1)}}{X_i^{(n-1)}}. \tag{S5}$$

The strain described in Eq. (S5) is referred to as the instantaneous true strain [S1]. From Eqs. (S3)–(S5), the three types of Poisson's ratios are described as follows:

$$\nu_{ij}^{\text{eng}} = -\frac{\varepsilon_j}{\varepsilon_i} \tag{S6}$$

$$\nu_{ij}^{\log} = -\frac{\varepsilon_j^{\log}}{\varepsilon_i^{\log}} \tag{S7}$$

$$\nu_{ij}^{\text{int}} = -\frac{\varepsilon_j^{\text{int}}}{\varepsilon_i^{\text{int}}} \tag{S8}$$

Equation (S8) is referred to as Poisson's function, which is analogous to the tangent stiffness at the current configuration. Poisson's function has a larger margin of error compared with the other Poisson's ratios such as Eq. (S6) or (S7), because experimental noise in $\varepsilon_i^{\text{int}}$ severely affects the Poisson's function. To avoid this difficulty, we calculated the median Poisson's function from four measured points and expressed the average function with polynomial fitting. Figure S5 indicates the data processing for the ν_{ij}^{int}-curves measured by the tensile tests for the nylon structure (see Fig. 2c). Note that the four-point median processing was skipped when calculating ν_{xy}^{int} for the outer cell because the raw data had satisfactory accuracy. The raw data of the Poisson's function out-of-plane (*x*-*z*) showed a wider variation

of errors than those in-plane (x-y). One of the main reasons is the different number of pixels between the images taken by the two types of the cameras.

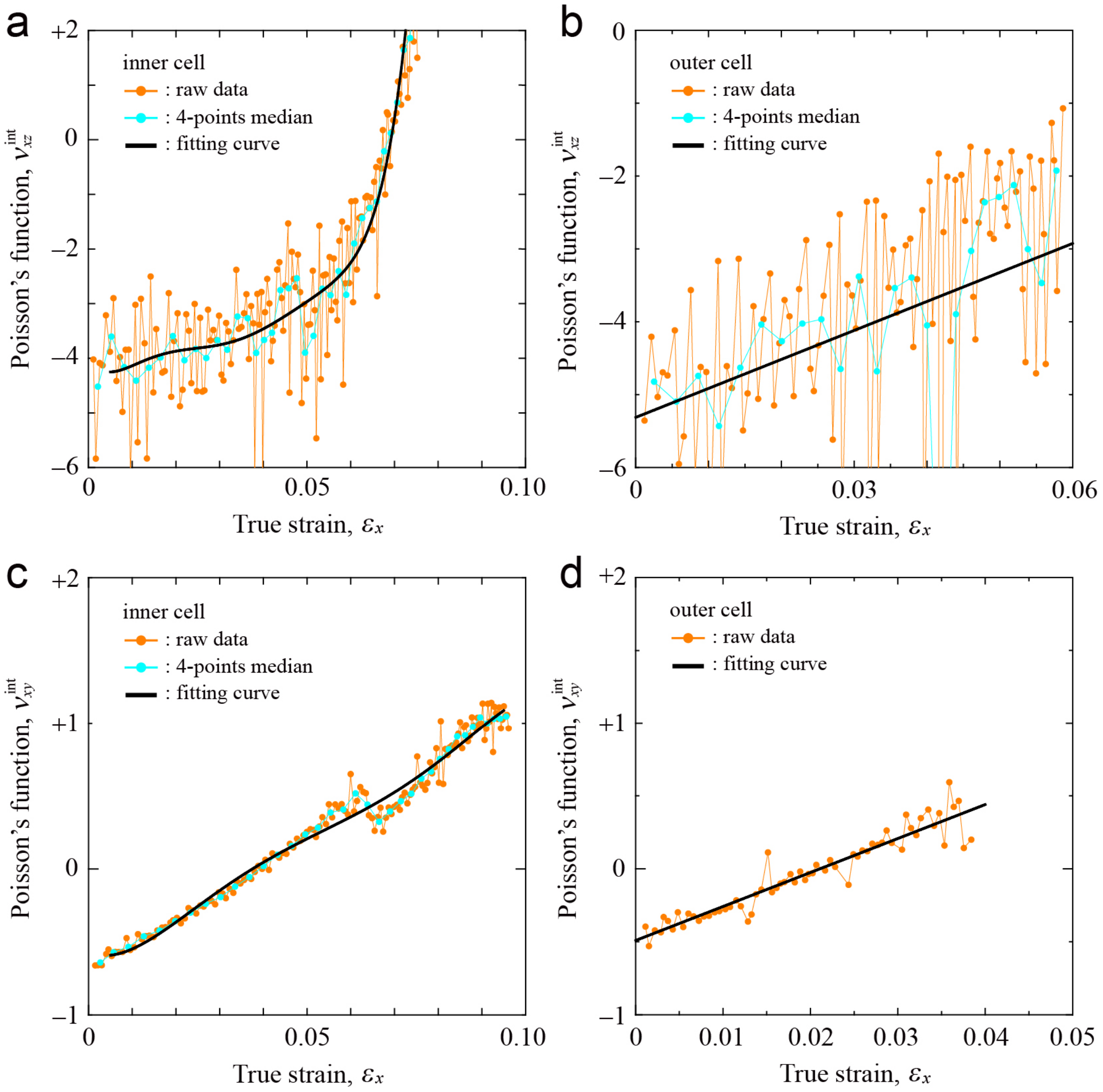

Fig. S5 Calculations of the Poisson's function ν_{ij}^{int} for nylon structure with median and fitting processing; (a) ν_{xz}^{int} for inner cell with a 6th-order polynomial fitting curve (b) ν_{xz}^{int} for outer cell with a linear fit (c) ν_{xy}^{int} for inner cell with the 5th-order polynomial fitting curve, (d) ν_{xy}^{int} for outer cell with a linear fit.

Supplementary Discussion 4: Finite Element Modeling of a Periodic Unit Cell

To obtain the elastic properties of the proposed periodic framework, we performed the finite element analyses using Abaqus (ver. 6.14-1, Dassault Systèms) [S2]. Figure S6 shows an overview of the finite element model. The unit cell consisted of beam and column components of the nylon material. The Young's modulus and Poisson's ratio were input as 1700 MPa and 0.40, respectively. All the boundary surfaces of the cell framework were connected with rigid plates, and were set with a Young's modulus a million times as large as that of nylon to impose multi-point constraints in a periodic fashion; all the displacements of the plates A and C at one side, not including ones in the major axes, were consistent with those of the plates at the other side. One side of the structural unit in each of the X_i-directions was fixed on the X_j-X_k plane and the four forces were applied to opposite sides in either direction (X_1-, X_2- or X_3-axis). In the mesh processing, the body was divided with triangular quadratic elements (Abaqus element code CPE6H), as shown in Fig. S6. To account for geometric nonlinearity the nonlinear finite-element code ABAQUS/STANDARD was selected to analyze the uniaxial deformation of the structure. This was necessary for representing the out-of-plane behaviors.

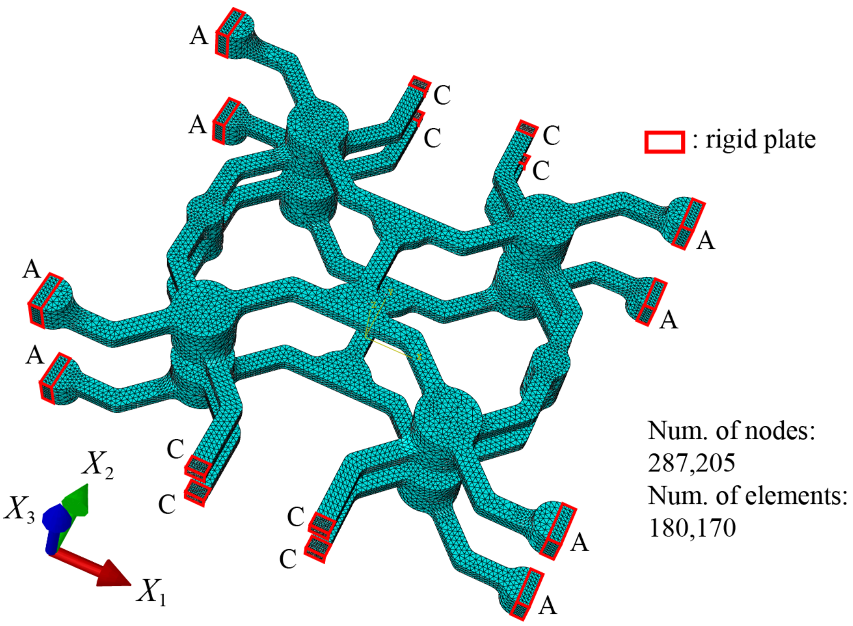

Fig. S6 Finite element model of a periodic unit cell under uniaxial deformation.

References for Supplementary Information:

[S1] C. W. Smith, R. J. Wootton, K. E. Evans, *Exp. Mech*. **39** (1999), 356.

[S2] ABAQUS, Version 6.14 User's Manual, Dassault Systèms Simulia Corp., Providence, RI, USA **2014**.

Captions for Supplementary Figures:

Figure S1:

Uniaxial tensile tests for the ABS microstructure pipes with or without silicone oil; (a) the CAD-data, (b) the uniaxial tensile testing, and (c) load–stain curves under uniaxial extension.

Figure S2:

Schematic of the upper jig attachment.

Figure S3:

Example showing the image processing steps for *x*-*z* surface image of ABS structure.

Figure S4:

Schematic of the camera parameters used to correct the dimensions of the specimen.

Figure S5:

Calculations of the Poisson's function ν_{ij}^{int} for nylon structure with median and fitting processing; (a) ν_{xz}^{int} for inner cell with a 6th-order polynomial fitting curve (b) ν_{xz}^{int} for outer cell with a linear fit (c) ν_{xy}^{int} for inner cell with the 5th-order polynomial fitting curve, (d) ν_{xy}^{int} for outer cell with a linear fit.

Figure S6:

Finite element model of a periodic unit cell under uniaxial deformation.

Captions for Supplementary Tables:

Table S1:

Information on as-prepared pipe specimens and test results.

Table S2:

Information on as-prepared cellular structure specimens and test results.

Captions for Supplementary Movies:

Movie S1:

Image sequence of out-of-plane (*x*–*z*) deformation of nylon resin structure under tensile loading. The stroke displacement per image is 0.25 mm. The movie has 159 images and the tension test was terminated at a displacement of 39.50 mm.

Movie S2:

Image sequence of in-plane (*x*–*y*) deformation of nylon resin structure under tensile loading, which is coincident with that of Movie S1.

Movie S3:

Image sequence of out-of-plane (*x*–*z*) deformation of the ABS resin structure under tensile loading in the third test. The stroke displacement per image is 0.25 mm. The movie has 61 images and the tension test was terminated at a displacement of 15.00 mm.

Movie S4:

Image sequence of out-of-plane (*x*–*z*) deformation of ABS resin structure under compressive loading. The stroke displacement per image is 0.25 mm. The movie has 29 images and the compression test was terminated at a displacement of 7.00 mm.
